# Separation of Copper and Nickel Metal Ions from Electroplating Wastewater by Ultrafiltration with Tartaric Acid and Sodium Citrate Reinforced Sodium Polyacrylate Complexation

**DOI:** 10.3390/membranes14110240

**Published:** 2024-11-14

**Authors:** Hanwen Zhang, Rui Cui

**Affiliations:** 1 College of Resources and Environmental Engineering, Wuhan University of Science and Technology, Wuhan 430081, China; zhw2000528yi@163.com; 2 Hubei Key Laboratory for Efficient Utilization and Agglomeration of Metallurgic Mineral Resources of Wuhan University of Science and Technology, Wuhan 430081, China

**Keywords:** PAAS, copper, nickel, tartaric acid, sodium citrate, complexes

## Abstract

In this study, sodium polyacrylate (PAAS) and ultrafiltration membranes were used to extract and separate Cu^2+^ and Ni^2+^ ions from electroplating wastewater. The effects of pH, the P/M ratio (mass ratio of sodium polyacrylate to metal ions), tartaric acid, and sodium citrate on the complexation of Cu^2+^ and Ni^2+^ by sodium polyacrylate were investigated. The retention of Cu^2+^ and Ni^2+^ by PAAS in single metal solutions with a P/M ratio = 4 and pH = 5 differed by 45.36%. When the complexation system of PAAS with a single metal contained tartaric acid and sodium citrate, the retention of PAAS for Cu^2+^ and Ni^2+^ increased to 80.36% and 58.84%. PAAS retention for Ni^2+^ decreased, but retention for Cu^2+^ remained the same. All the results indicated that there was competition between tartaric acid, sodium citrate, and PAAS for the adsorption of Cu^2+^ and Ni^2+^. Some of the Ni^2+^ complexed with PAAS were detached from PAAS complexed by tartaric acid and sodium citrate and permeated through the membrane pores, while the Cu^2+^ complexed with PAAS was not complexed by tartaric acid and sodium citrate and could not permeate through the membrane pores. Therefore, this study helps to provide a theoretical basis for the separation of Cu^2+^ and Ni^2+^ in electroplating wastewater.

## 1. Introduction

Currently, many electroplating wastewater treatments typically rely on traditional chemical treatment methods. After meeting the discharge standards, there is little recycling of the wastewater. Electroplating wastewater with low concentrations of metal ions is a source of acidic wastewater that has been less studied. This type of wastewater is also rich in heavy metals and sulfates [1]. Electroplating chromium on thermoplastics requires a multilayer electrodeposition treatment with several interim rinsing steps [2]. Heavy metals such as chromium, copper, zinc, nickel, and iron typically present in electroplating wastewater pose a threat to both human health and the environment even at very low concentrations as a result of their high toxicity and potential for bioaccumulation [3]. The World Health Organization reports that a long-term intake of these toxic metals can cause significant damage to various organs of the body [4]. Among them, copper ions in electroplating wastewater are mainly in the forms of copper sulfate, copper pyrophosphate, and cuprous cyanide, etc.; nickel ions are mainly in the forms of nickel sulfate, nickel chloride, phosphite, borate, suspended solids, etc. Due to the similarity in the physical and chemical properties of copper and nickel, it is more difficult to separate Cu^2+^ and Ni^2+^ when they co-exist in electroplating wastewater. There are various treatment methods such as co-precipitation, electrochemical deposition, ion exchange, adsorption, flotation, crystallization, and membrane filtration, as well as combinations of different treatment methods [5]. Chemical precipitation technology is commonly used for separating and recovering Cu^2+^ and Ni^2+^ from electroplating wastewater [6]. However, this method has several drawbacks. It is difficult to remove subsequent ammonia nitrogen and total phosphorus [7], and it generates a large amount of sludge during the treatment process [8], increasing the treatment cost. Moreover, some metal ions are lost during the treatment process, resulting in poor treatment capabilities for low-concentration electroplating wastewater. Zohaib Abbas et al. [9] pointed out that graphene and its modified materials can adsorb heavy metal ions well. This is because graphene has an ordered and structured form, a high specific surface area, and flexible surface functionalization options. However, graphene and its modifications are costly and not very selective for metal ions. Therefore, to solve the problem of recovering Cu^2+^ and Ni^2+^ from electroplating wastewater, it is crucial to use more environmentally-friendly production processes and advanced wastewater treatment technologies [10] that meet these requirements.

Membrane processing has proven to be a key technology involving separation and purification [11]. Among these, complexation–ultrafiltration technology has multiple advantages, such as energy conservation, environmental protection, simple processes, convenient operation, a small footprint, a better separation effect [12,13], and the ability to recover macromolecules [14]. Complex ultrafiltration technology utilizes selectively permeable membranes to separate, classify, purify, and concentrate mixtures. This process is driven by external energy or chemical potential differences [15]. The process where metal ions complex with complexing agents to form macromolecular complexes that are retained by ultrafiltration membranes is termed complexation–ultrafiltration [16]. At present, several water-soluble macromolecular complexing agents, such as sodium polyacrylate (PAAS), polyetherimide (PEI), and acrylic acid–maleic acid copolymer (PMA-100), have been utilized for dealing with heavy metal ions in wastewater [17]. Zhang X J et al. [18] used PEI for the complexation–ultrafiltration of Ni^2+^ to investigate the effect of the polymer-to-metal ion mass ratio (P/M ratio) and pH on the retention rate of Ni^2+^, and the results indicated that the retention rate of Ni^2+^ increased with the increase in the P/M ratio or pH. Camarillo R et al. [19] used polyacrylic acid (PAAS) complexation–ultrafiltration for the treatment of Ni^2+^, Cu^2+^, and Zn^2+^ in primary battery wastewater, and the retention rate of the three metal ions could reach 85–97%. Poly(sodium acrylate) (PAAS) is a polymer with remarkable water solubility and a high affinity for heavy metal ions. It is widely used as a complexing agent for heavy metal treatment and has strong applicability. Different complexation conditions apply to various metal ions. Currently, the membrane treatment technology for electroplating wastewater mainly focuses on the direct removal of heavy metals, resulting in the transfer of pollutants and waste resources. However, there is a lack of research on the classification and recovery of heavy metals in wastewater [10]. Most commonly employed commercial metal ion carriers in membrane techniques can facilitate the effective separation of these ions; however, their selectivity is relatively low [20]. PAAS can be complexed with Cu^2+^ and Ni^2+^ to a certain extent. Nevertheless, the disparity in the complexation of PAAS for Cu^2+^ and Ni^2+^ is negligible. Therefore, it is essential to discover a method to enhance the complexation difference in PAAS for metal ions.

Some materials with adsorption and complexation functions have been used in ultrafiltration membranes for the removal of metal ions from soil. Arfa Iqbal et al. noted that polymer nanocomposite membranes have great potential for removing heavy metal ions [5]. By introducing nanomaterials into the filter membrane, the effect of polymers on heavy metal ions can be changed through the adsorption of nanomaterials. Jun Zhu et al. pointed out that the presence of small anionic ligands could stimulate the sorption of heavy metals on soil constituents through the electrostatic effect, the formation of ternary complexes, and surface precipitates [21]. However, heavy metal sorption can also be inhibited by small anionic ligands through the formation of soluble complexes. These ligands compete with heavy metals for sorption sites, suppress the formation of surface precipitates, and dissolve the sorbent, among other factors. Xue ji You et al. [22] investigated that EDTA can, by complexing with heavy metal ions, reduce the adsorption capacity of soil minerals for heavy metals by decreasing the specific surface area of soil minerals and blocking the active sites [23]. Previously, studies on low-molecular-weight anionic ligands have mainly focused on the effect of the soil adsorption of metal ions. However, further investigations on colloidal complexation for the ultrafiltration separation of metal ions are requisite.

To enhance the separation of diverse metal ions using complexation–ultrafiltration technology [24], most researchers focused on exploring the impacts of the complexing agent type, pH, the polymer-to-metal mass ratio (P/M ratio), and background electrolytes on the removal efficiency of various metal ions. Raffaele Molinari et al. showed that the selective separation of Cu^2+^ and Ni^2+^ can be achieved by adjusting pH and the P/M ratio [25]. Recently, some studies on shear have also been carried out. Qiang Zhang et al. [26] investigated the selective separation of Ni^2+^ and Cr^3+^ by rotating disk membrane complexation–ultrafiltration. However, its operation is not simple. It requires dealing with the distribution of the solution system, reducing the ratio of polymer to metal ions, or providing more power. The introduction of tartaric acid and sodium citrate may be able to improve the separation of Cu^2+^ and Ni^2+^. The main sources of tartaric acid and sodium citrate are plant root secretion, soil organic matter decomposition, and microbial metabolism products, which are lower in price, easier to obtain, and will not have a large impact on the environment when applied to electroplating wastewater treatment and subsequent discharge. And the actual plating wastewater composition is complex, as there may be a small amount of sodium citrate, the tartaric acid, compared to changing the pH and P/M ratio; in the same pH and P/M ratio conditions, to add a certain concentration of tartaric acid and sodium citrate is more convenient and economical.

The obvious difference between tartaric acid/sodium citrate and PAAS lies in their ability to undergo the Tyndall effect. The Tyndall effect is a characteristic of colloids. In a colloid, the particles in the dispersed phase range in size from approximately 1 to 100 nm. In contrast, a solution of tartaric acid/sodium citrate contains only molecules or ions with particle sizes of less than 1 nm, which makes it difficult to produce the Tyndall effect. Introducing tartaric acid and sodium into the complexation–ultrafiltration system to alter the binding state of metal ions and the macromolecular complexing agent has been considered. The complexation differences between tartaric acid, sodium citrate, the macromolecular complexing agent, and metal ions, as well as the particle size differences in tartaric acid molecules and the macromolecular complexing agent for the separation of metal ions has also been utilized. Moreover, the direct contact between the biomass and the membrane will lead to membrane contamination [11] and affect the efficiency of the membrane, but the particle size of tartaric acid and sodium citrate is much smaller than the membrane pores and the membrane contamination that can be caused is limited.

In this paper, the study focused on Cu^2+^ and Ni^2+^ in electroplating wastewater. Sodium polyacrylate (PAAS) served as the complex, and the polyethersulfone flat-plate ultrafiltration membrane was used as the separation medium. Through complexation–ultrafiltration experiments with monometallic systems, the effect of tartaric acid/sodium citrate on the separation of Cu^2+^ and Ni^2+^ by PAAS was investigated, and membrane contamination was monitored. Additionally, the mechanism of action of tartaric acid and sodium citrate in enhancing the separation of Cu^2+^ and Ni^2+^ metal ions was analyzed by chemical calculations, infrared spectroscopic analysis, and XPS of the solution system.

## 2. Experimental Section

### 2.1. Membranes

The laboratory uses tubular ultrafiltration membranes for the pretreatment of PAAS and flat membranes for subsequent ultrafiltration after metal ions bind to PAAS and small-molecule complexing agents. Due to the long working time and continuous work for the pretreatment of PAAS, the tubular ultrafiltration membrane is used. The price of the flat membrane is lower than that of the tubular ultrafiltration membrane, and it is more convenient to replace and clean and can be synchronized with multiple groups, so the flat membrane was used in the subsequent condition experiments. The ultrafiltration membrane selection is shown in Table 1.

### 2.2. Experimental Chemicals and Solutions

Sodium polyacrylate with a molecular weight of 30,000 kDa was selected as the macromolecular complexing agent. NaOH and H₂SO₄ were chosen as pH-adjusting agents. NiSO_4_∙6H_2_O and CuSO_4_ were used as raw materials for simulating plating wastewater, and tartaric acid and sodium citrate were used as small-molecule complexing agents affecting complexation. The solutes of NiSO_4_∙6H_2_O, CuSO_4_, tartaric acid, and sodium citrate were directly added into deionized water and stirred to dissolve. However, sodium polyacrylate requires heating and stirring to dissolve. In this study, the test water was deionized and ultrapure water was used to configure the metal ion standard solution. The test drugs used are shown in Table 2.

### 2.3. Analytical Methods

PAAS concentration was measured by a UV spectrophotometer. Firstly, different concentrations of PAAS pure solutions were prepared. Then, these PAAS solutions were scanned by a UV-2550 ultraviolet–visible spectrophotometer (SHIMADZU, Kyoto, Japan) for the full spectrum. There was a certain corresponding relationship between the C=O content in PAAS and the absorbance of the solutions to some extent. The UV spectra showed that the PAAS solution had a strong absorption peak at 207 nm. Therefore, it was possible to photometrically measure the PAAS content of different PAAS solutions at 207 nm by using the correspondence between the absorbance of the solution and the PAAS concentration. All the analyses were performed three times to obtain the average value.

Before ultrafiltration, the metal ion concentration in the ultrafiltration cup is fixed. By detecting the metal ion concentration in the permeate, the metal ion concentration in the retention solution can be calculated. The concentrations of Cu^2+^ and Ni^2+^ in the permeate and retentate were determined by novAA350 atomic absorption spectrophotometry (Analytik Jena, Jena, Germany).

At pH = 5, a PAAS/small-molecule complexing agent with Cu^2+^ and Ni^2+^ metal ions had a mass ratio of 4; the solutions were irradiated with a laser pointer, and the particle size distributions of several mixtures were measured by a ZetasizerNano zeta potential meter (ZS90, Malvern, UK).

The mixed solution of PAAS and metal ions was ultrafiltered. The gel on the ultrafiltration membrane was collected and dried in a vacuum drying oven. The dried samples were then ground using a mortar and pestle. Meanwhile, the mixed solution of tartaric acid, sodium citrate, and metal ions was frozen in liquid nitrogen and subsequently freeze-dried. To investigate the variations in the functional groups of PAAS, tartaric acid, and sodium citrate, the Fourier transform infrared spectra (FTIR) of the milled samples were obtained by compressing them with the KBr disk method. The samples were scanned 32 times on an infrared spectrometer (Nicolet iS50, Thermo Fisher Scientific, Shanghai, China) at a resolution of 4 cm^−1^ within the spectral range of 400 to 4000 cm^−1^.

Samples of CuSO_4_, NiSO_4_∙6H_2_O, PAAS complexed with Cu^2+^, PAAS complexed with Ni^2+^, tartaric acid complexed with Cu^2+^, tartaric acid complexed with Ni^2+^, sodium citrate complexed with Cu^2+^, and sodium citrate complexed with Ni^2+^ were prepared by a sampling method similar to the FTIR spectroscopy test preparation. Then, the peaks of the metal ions were fitted to the peaks using a Thermo Scientific K-Alpha X-ray photoelectron spectrometer. A split peak was fitted to the metal ions to observe the shift in the Cu^2+^ and Ni^2+^ absorption peaks.

### 2.4. PAAS Pretreatment Test

The water-soluble polymer PAAS has an average molecular weight ranging from hundreds of thousands to tens of millions of Daltons, which is much greater than the cut molecular weight of the PES ultrafiltration membrane. However, the molecular weight distribution of PAAS is not uniform, with short-chain PAAS having small molecular weights.

A pretreatment system was used to remove the low-molecular-weight PAAS. Driven by a peristaltic pump, low-molecular-weight PAAS and water molecules permeated through the ultrafiltration membrane and flowed out, while the unpermeated large-molecule PAAS flowed back to the stock tank.

The refill tank was sealed to keep the reserve tank level stable. When the liquid level in the barrel dropped to the bottom of the inlet pipe, the outlet pipe addded deionized water to the barrel until the liquid level ascended to the bottom of the inlet pipe. At an initial PAAS concentration of 1000 mg/L and a temperature of 25 °C, the effect of the recharge water volume on the retention of low-molecular-weight PAAS in the solution was investigated. With the increase in the recharge water volume, the removal of low- and medium-molecular-weight PAAS from PAAS exceeded 99%. The pretreatment test setup is shown in Figure S1 of the Supplementary Materials.

### 2.5. Complexation–Ultrafiltration Test

A 1000 mg/L Cu^2+^ solution, 1000 mg/L Ni^2+^ solution, a certain concentration of pretreated PAAS solution, 50 × 10^−3^ mol/L tartaric acid solution, and 50 × 10^−3^ mol/L sodium citrate solution were prepared. PAAS, metal ions, tartaric acid, and sodium citrate were added following different P/M ratios (the mass ratio of PAAS to metal ions). After thorough mixing, the pH of the solution system was adjusted. Subsequently, the solution was conveyed to the ultrafiltration system for ultrafiltration.

**Figure 1:**
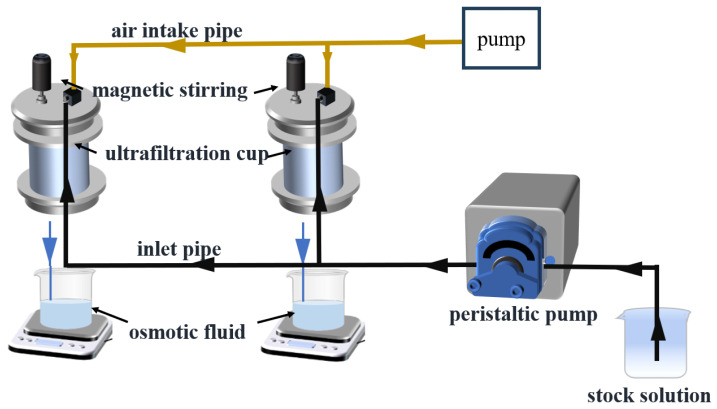
Diagram of ultrafiltration test setup.

The ultrafiltration device is shown in Figure 1. First, the slurry was conveyed into the ultrafiltration cup using the peristaltic pump. Next, the ultrafiltration cup was sealed with a fixture. After that, the air pump was employed to deliver pressure to the ultrafiltration cup while the magnetically coupled shaft stirrer was used to stir the slurry. Subsequently, the preset pressure gauge was adjusted to control the system pressure at 0.15 MPa. When 30 mL of permeate flowed out and the pressure stabilized the dead volume of the ultrafiltration membrane pores was removed. Finally, 100 mL of the filtered permeate was taken to determine the concentrations of Cu^2+^ and Ni^2+^ by atomic flame absorption spectrometry. The time required for 100 mL of permeate to permeate was recorded for the calculation of the membrane flux.

The retention effect of PAAS on Cu^2+^, Ni^2+^, and the membrane flux was determined by Formulas (1)–(3).

(1) $$R=1\;-\frac{C_{M}}{C_{m}}$$

(2) $$Q=R_{C}\;-R_{N}$$

(3) $$J_{t}=\frac{V}{A\times t}$$

In the formula, the parameters are defined as follows:

R—metal ion retention rate, %;
C_M_—metal ion concentration in permeate, mg/L;
C_m_—metal ion concentration in stock solution, mg/L;
R_C_—Cu^2+^ ion concentration in permeate, mg/L;
R_N_—Ni^2+^ concentration in permeate, mg/L;
Q—Cu^2+^-Ni^2+^ retention rate difference, %;
J_t_—membrane flux, mL·m^-2^·s^-1^;
V—permeate volume, mL;
A—ultrafiltration membrane surface area, m^2^;
t—permeation time, s.

## 3. Results and Discussion

### 3.1. Particle Size Analysis

**Figure 2:**
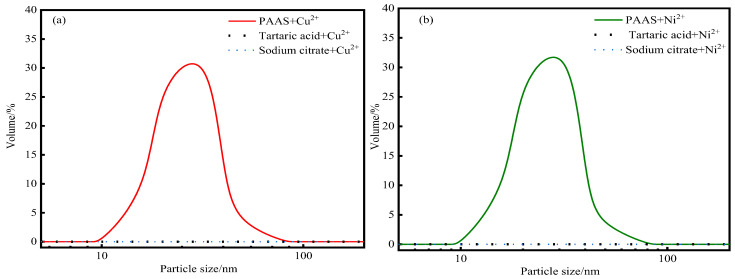
Particle size distribution. (**a**) Cu^2+^ system, (**b**) Ni^2+^ system. (Temperature = 20 °C, pH = 5, P/M ratio = 4.)

When the solution was irradiated with a laser pointer, no visible column of light appeared in the mixed solution of tartaric acid, sodium citrate, and metal ions. On the contrary, the Tyndall effect appeared in the mixed solution of PAAS and metal ions. In the Supplementary Materials, Figure S2 illustrates the phenomenon of laser irradiation. The results of particle size testing, shown in Figure 2a,b, show that the substances within the mixed sample of tartaric acid/sodium citrate and metal ions had no distribution in the particle size range of 10–100 nm, while the mixed sample of PAAS and metal ions had a volume share of 30–40% of the substances with a particle size of 10–100 nm. The particle size distribution is shown in Figure 2.

In complex ultrafiltration technology, the membrane pores allow water molecules and small molecules to pass through, while substances with a particle size larger than the membrane pores are retained on the feed side of the membrane [27,28]. Therefore, it can be preliminarily inferred that in the system of PAAS, metal ions, and small-molecule complexing agents, if the metal ions are complexed by PAAS, they will be retained by the ultrafiltration membrane; if the metal ions are complexed by the small-molecule complexing agent or are not complexed, they will be able to pass through the ultrafiltration membrane.

### 3.2. Tests of P/M Ratio and pH Conditions

**Figure 3:**
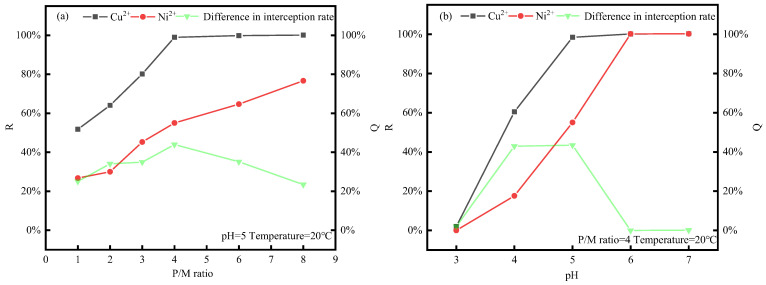
Variation in Cu^2+^-Ni^2+^ retention rate with P/M ratio and pH. (**a**) Effect of P/M ratio on retention rate. (**b**) Effect of pH on retention rate.

The effect of the P/M ratio (PAAS-to-metal ion mass ratio) on the behavior of the PAAS complexation of Cu^2+^ and Ni^2+^ and membrane contamination was investigated at pH = 5 and temperature = 20 °C. Additionally, the effect of pH on the behavior of the PAAS complexation of Cu^2+^ and Ni^2+^ was investigated at P/M ratio = 4. The experimental results are shown in Figure 3a,b.

As can be seen from the results in Figure 3a,b, the retention rate of PAAS for both Cu^2+^ and Ni^2+^ increased with the increase in the P/M ratio and pH. When the P/M ratio [29] increased from one to four, the difference between the retention rates of Cu^2+^ and Ni^2+^ increased from 25.06% to 45.36%. The pH plays a key role in determining the strength of electrostatic interactions, which significantly affects the complexation between the two biopolymers [30]. When the pH increased to five, the difference between the retention rates of Cu^2+^ and Ni^2+^ by PAAS increased from 0% to 45.36%. With further increases in the P/M ratio and pH, the retention rates of PAAS for both metal ions increased, while the difference between the retention rates of PAAS for the two metal ions gradually decreased, which is consistent with Jing Gao’s study [31]. This may be because at higher P/M ratio and pH conditions, PAAS is less protonated and can provide more complexation sites for Cu^2+^ and Ni^2+^, but Cu^2+^ and Ni^2+^ may have different complexation states with PAAS in the same state. The results of the complexation–ultrafiltration test showed that the difference between the retention rates of the two metal ions was the largest under the conditions of P/M ratio = 4 and pH = 5.

As can be seen from the results in Figure S3a,b, after the increase in the P/M ratio, the membrane flux did not show more obvious fluctuations. This is similar to Kwang’s results [32]. Kwang’s study showed that increasing the amount of polymer does not reduce the membrane flux of UF membranes and that the membrane flux does not show significant fluctuations after increasing the pH. Moreover, under the conditions of P/M ratio = 4 and pH = 5, the membrane flux is at the normal average level, which does not affect the system operation.

### 3.3. Ultrafiltration Test of a Single Metal Ion System with Tartaric Acid Complexation

#### 3.3.1. Test of Tartaric Acid Concentration Condition

**Figure 4:**
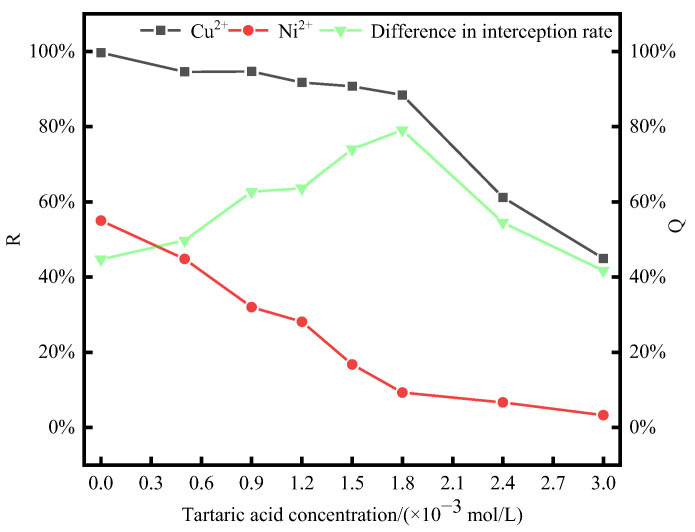
The effect of tartaric acid concentration on the retention rate. (P/M ratio = 4; pH = 5; temperature = 20 °C.)

Under the previously discussed optimum conditions (P/M ratio = 4, pH = 5, and temperature of 20 °C) for the separation of the two metal ions, PAAS was first mixed and blended sufficiently with the metal ions. Then, different concentrations of tartaric acid were added to PAAS. The effect of tartaric acid concentration on the complexation behavior of Cu^2+^ and Ni^2+^ by PAAS was investigated, and the experimental results are shown in Figure 4.

From the results in Figure 4, it can be seen that under the conditions of P/M ratio = 4, pH = 5, and a temperature of 20 °C, the retention rate of PAAS for Cu^2+^ varied less when the concentration of tartaric acid ranged from 0 to 1.8 × 10^−3^ mol/L. This might be because after adding a small amount of tartaric acid, Cu^2+^ could be complexed with tartaric acid and PAAS simultaneously or Cu^2+^ was complexed by PAAS, so the Cu^2+^ retention rate did not decrease significantly. However, after the concentration of tartaric acid increased, the retention rate of PAAS for Cu^2+^ decreased more obviously. When the concentration of tartaric acid was in the range of 0 to 1.8 × 10^−3^ mol/L, the retention rate of Ni^2+^ by PAAS varied significantly. This might be because a portion of Ni^2+^ was separated from PAAS and combined with tartaric acid to pass through the membrane pore, causing a decline in the retention rate of Ni^2+^ by PAAS. As the concentration of tartaric acid increased, most of the Ni^2+^ had passed through the ultrafiltration membrane, and the retention rate of Ni^2+^ by PAAS gradually approached zero. When no tartaric acid was added, the difference between the PAAS retention rates for Cu^2+^ and Ni^2+^ was only 45.36%. However, when the tartaric acid concentration was increased to 1.8 × 10^−3^ mol/L, the difference between the retention rates of these two metal ions reached a maximum of 80.36%. As the amount of tartaric acid continued to increase, the complexation of Cu^2+^ by PAAS was inhibited, and the difference in retention between the two metal ions decreased. After the addition of tartaric acid, there may be competition for adsorption in the system [33,34,35]. Therefore, a certain concentration of tartaric acid can widen the gap in the retention rate of PAAS for Cu^2+^ and Ni^2+^, which is conducive to the separation of these two metal ions.

Tartaric acid may contaminate the ion exchange membrane [36]. As was described in the previous section, tartaric acid molecules can pass through the ultrafiltration membrane pores and are less likely to contaminate the ultrafiltration membrane. Thus, after the addition of tartaric acid, the membrane flux of the ultrafiltration membrane was small. The changes in membrane flux are shown in Figure S3c of the Supplementary Materials.

#### 3.3.2. Influence of Solution System pH on Tartaric Acid-Enhanced Separations

**Figure 5:**
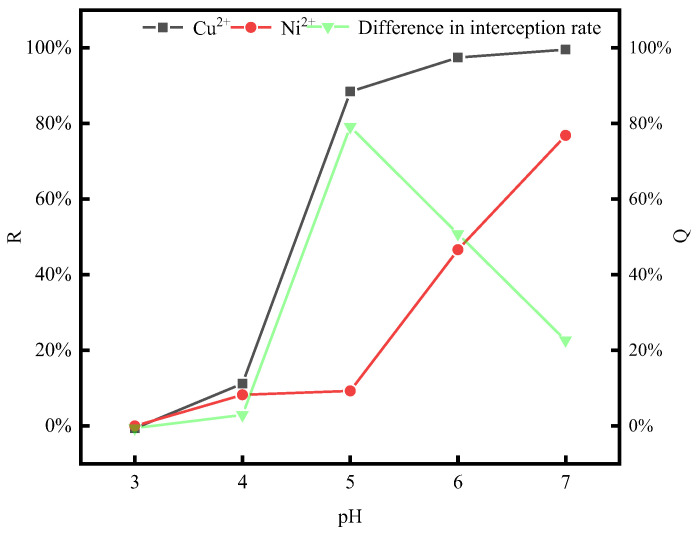
The effect of pH on the retention rate after the addition of tartaric acid. (P/M ratio = 4; temperature = 20 °C. [Tartaric acid concentration] = 1.8 × 10^−3^ mol/L.)

Under the optimal conditions where P/M ratio = 4, the tartaric acid concentration is 1.8 × 10^−3^ mol/L, and the temperature is 20 °C, PAAS and metal ions were thoroughly mixed with sufficient stirring. Subsequently, tartaric acid was added to bring the tartaric acid concentration of the solution system to 1.8 × 10^−3^ mol/L. After that, the pH was adjusted using a pH-adjusting reagent to study the influence of the solution system’s pH on the enhanced separation effect of tartaric acid. The influence of pH on the tartaric acid-strengthening separation was investigated, and the results are presented in Figure 5.

From the results in Figure 5, it can be seen that under the conditions of P/M ratio = 4 and a tartaric acid concentration of 1.8 × 10^−3^ mol/L, the retention rate of PAAS for Cu^2+^ and Ni^2+^ increases as the pH rises. At pH = 3, tartaric acid and PAAS do not form complexes with Cu^2+^ and Ni^2+^ [31,37]. When the pH is increased to five, the retention rate of PAAS for Cu^2+^ rises significantly, while the increase for Ni^2+^ is less. For Ni^2+^, the retention rate of PAAS increases more significantly for Cu^2+^, while the increase for Ni^2+^ is less. It may be because after the pH is elevated, the reduced protonation of PAAS enables it to complex with Cu^2+^ [14] and the PAAS complexes with Cu^2+^ generated in the system are more stable than the tartaric acid complexes with Cu^2+^. As the pH continues to increase, the PAAS retention rate for Cu^2+^ shows a slower increasing trend, while that for Ni^2+^ increases more significantly. It may be that the Ni^2+^ in the Ni^2+^ system requires more complexation sites. Thus, it is necessary to keep increasing the pH to provide more PAAS complexation sites. In contrast, the Cu^2+^ in the Cu^2+^ system requires fewer complexation sites, so PAAS can complex with most of the Cu^2+^ at pH = 5. Moreover, the difference between the retention rates of the two metal ions is the largest at pH = 5.

After changing the pH, a small effect of pH on the membrane flux was observed before the addition of tartaric acid (see Figure S3b in the Supplementary Materials). After adding tartaric acid, the change in the morphology of tartaric acid had a minor impact on the membrane flux of the UF membranes. The changes in the membrane flux are shown in Figure S3d of the Supplementary Materials.

### 3.4. Ultrafiltration Test of a Single Metal Ion System with Sodium Citrate Complexation

#### 3.4.1. Test of Sodium Citrate Concentration Condition

**Figure 6:**
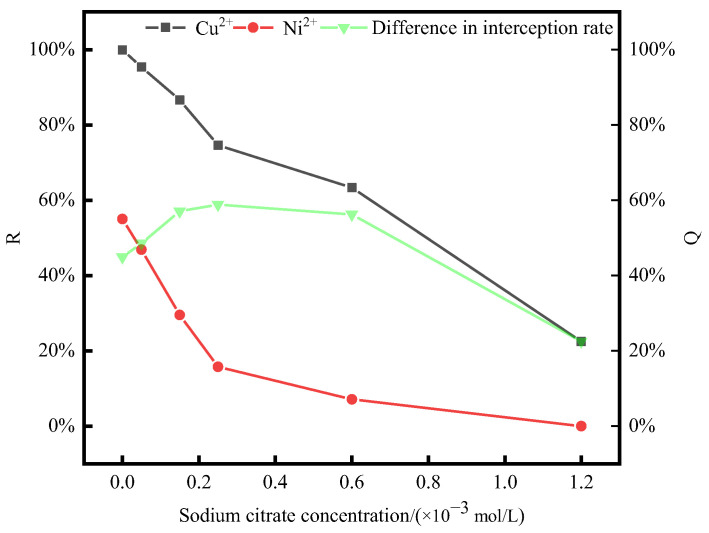
The effect of sodium citrate concentration on the retention rate. (P/M ratio = 4; pH = 5; temperature = 20 °C.)

Under the previously discussed optimum conditions (P/M ratio = 4, pH = 5, and temperature of 20 °C) for the separation of two metal ions, PAAS was first added to different concentrations of sodium citrate after being thoroughly mixed with the metal ions. The influence of sodium citrate concentration on the complexation behavior of PAAS with Cu^2+^ and Ni^2+^ was explored, and the experimental results are presented in Figure 6.

From the results in Figure 6, it can be seen that under the conditions of P/M ratio = 4, pH = 5, and a temperature of 20 °C, when the concentration of sodium citrate was 0–0.25 × 10^−3^ mol/L compared with PAAS for Ni^2+^, the changing trend of the retention rate of PAAS for Cu^2+^ was relatively flat. This may be caused by the competitive complexation of sodium citrate, PAAS, Cu^2+^, and Ni^2+^ in the system. After adding sodium citrate, there were fewer complexation sites for Cu^2+^ than for Ni^2+^. Moreover, the complexation sites of sodium citrate that can complex with Cu^2+^ are less than those for Ni^2+^. After adding sodium citrate, the amount of Cu^2+^ complexed to sodium citrate is less than that of Ni^2+^ complexed to sodium citrate. After increasing the concentration of sodium citrate, the retention rate of PAAS for Cu^2+^ decreased significantly. When the concentration of sodium citrate ranged from 0 to 0.25 × 10^−3^ mol/L, the retention rate of PAAS for Ni^2+^ varied greatly. After increasing the concentration of sodium citrate, most of the Ni^2+^ had already passed through the ultrafiltration membrane, and the retention rate of PAAS for Ni^2+^ tended to be 0. When sodium citrate was not added, the difference between the retention rates of Cu^2+^ and Ni^2+^ was only 45.36%. When sodium citrate was added at a concentration of 0.25 × 10^−3^ mol/L, the difference between the retention rates of the two metal ions reached a maximum of 58.84%. Continuing to increase the dosage of sodium citrate, PAAS complexation with Cu^2+^ was also strongly inhibited, and the difference between the retention rates of the two metal ions decreased. After adding sodium citrate, there was competition for complexation in the system [38,39]. Therefore, a certain concentration of sodium citrate can widen the gap in the retention rate of PAAS for Cu^2+^ and Ni^2+^, which is conducive to the separation of these two metal ions.

As can be seen in Figure S3e of the Supplementary Materials, after the addition of sodium citrate, the ultrafiltration membrane’s membrane flux was small. Moreover, as shown in the previous sections, sodium citrate molecules could pass through the pores of the ultrafiltration membrane. Consequently, the addition of sodium citrate had a relatively small effect on the ultrafiltration membrane’s membrane flux.

#### 3.4.2. Influence of Solution System pH on Sodium Citrate-Enhanced Separations

**Figure 7:**
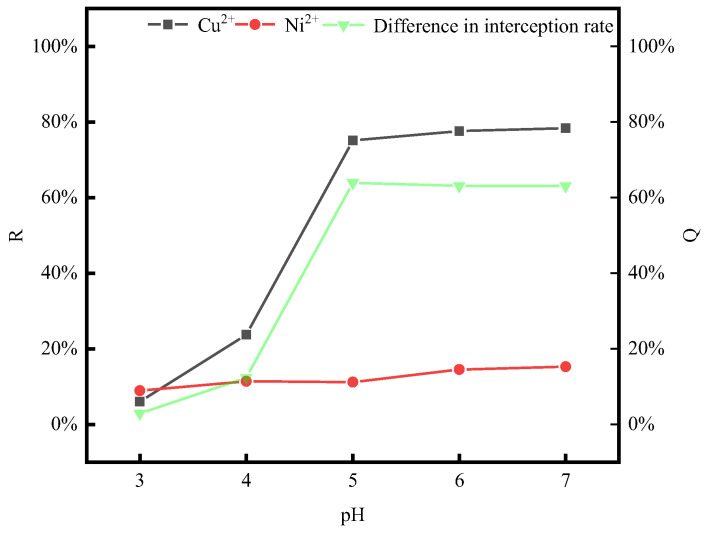
The effect of pH on the retention rate after the addition of sodium citrate. (P/M ratio = 4; temperature = 20 °C; sodium citrate concentration = 0.25 × 10^−3^ mol/L.)

Under the optimal conditions for the separation of the two metal ions (P/M ratio = 4, sodium citrate concentration of 0.25 × 10^−3^ mol/L, and temperature of 20 °C), PAAS and the metal ions were first mixed with sufficient agitation. Subsequently, sodium citrate was added to reach a concentration of 0.25 × 10^−3^ mol/L in the solution system. The pH-adjusting reagent was used to change the pH of the solution system, aiming to investigate the effect of pH on the enhanced separation. Also, the effect of the solution system pH on the enhanced separation effect of sodium citrate was investigated, and the experimental results are shown in Figure 7.

From the results of Figure 7, it can be seen that under the conditions of P/M ratio = 4 and a sodium citrate concentration of 0.25 × 10^−3^ mol/L, the retention rate of PAAS for Cu^2+^ and Ni^2+^ increased with the increase in pH. When pH = 3, PAAS did not complex with Cu^2+^ and Ni^2+^ [31,37]. Moreover, when the sodium citrate concentration was 0.25 × 10^−3^ mol/L, the retention rate of PAAS for Cu^2+^ increased significantly. However, when the sodium citrate concentration was increased, the retention rate of Ni^2+^ increased less. This may be because after the elevation of pH, the complexation sites of PAAS with Cu^2+^ and Ni^2+^ emerged. For Cu^2+^, the increase in the PAAS retention rate is more obvious, while for Ni^2+^, it is smaller. This may be because after the increase in pH, both PAAS and sodium citrate seem to be able to complex with Cu^2+^ and Ni^2+^, but the complexation of PAAS with Cu^2+^ is more stable and in the system, the complexation of sodium citrate with Ni^2+^ is more stable. When continuing to increase the pH, the growth trend in the retention rate of PAAS for Cu^2+^ and Ni^2+^ became slower. This was probably because there were fewer complexing sites required for Cu^2+^ in the Cu^2+^ system. At pH = 5, PAAS could complex with most of the Cu^2+^. In the Ni^2+^ system, sodium citrate provided complexing sites that could already complex most of the Ni^2+^. After the pH increase, although PAAS could also provide complexation sites for Ni^2+^, sodium citrate complexed with Ni^2+^ more stably, and most of the Ni^2+^ in the system was complexed with sodium citrate. The difference between the retention rates of the two metal ions was the largest at pH = 5.

After changing the pH, a small effect of pH on the membrane flux was observed before the addition of sodium citrate (Figure S3b in the Supplementary Materials). After the addition of tartaric acid, the change in the morphology of tartaric acid had a small effect on the UF membrane flux. The changes in the membrane flux are shown in Figure S3f of the Supplementary Materials.

### 3.5. Analysis of Solution Physicochemical Properties

In the complexation system, at a low pH, PAAS is highly protonated. As a result, Cu^2+^ and Ni^2+^ cannot be complexed with PAAS for retention. Under higher pH conditions, Cu^2+^ and Ni^2+^ will generate hydroxide precipitation. This will not only change the retention effect but also cause a certain degree of membrane contamination. Additionally, tartaric acid and sodium citrate dissociate different ligands under different conditions, and the component distribution within the solution varies significantly. The stabilization constants of different ligands bound to different metal ions also vary greatly. Therefore, it is necessary to analyze the hydrolysis and dissociation of Cu^2+^ and Ni^2+^, as well as tartaric acid and sodium citrate, under specific solution conditions.

#### 3.5.1. Metal Ion Morphological Analysis

**Figure 8:**
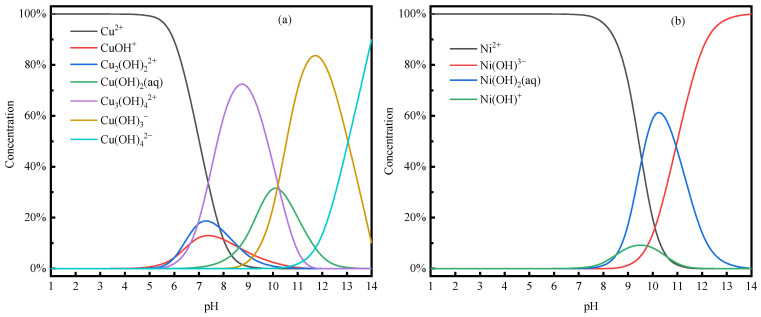
Distribution of metal ion solution components. (**a**) Cu^2+^ system, (**b**) Ni^2+^ system. ([Ni^2+^] = [Cu^2+^] = 10 mg/L; ionic strength: 0.001 mol/L; temperature: 20 °C.)

This test was carried out for plating wastewater containing a low concentration of metal ions. The maximum concentration of Cu^2+^ and Ni^2+^ in the wastewater was 10 mg/L. Visual MINTEQ 4 software was utilized to simulate and calculate the chemical forms of Cu^2+^ and Ni^2+^ solutions with a concentration of 10 mg/L under the condition of pH values ranging from 1 to 14, and the results are presented in Figure 8.

From Figure 8a,b, it can be observed that in the pH range of 1–5, the Cu^2+^ and Ni^2+^ in the solution existed as Cu^2+^ and Ni^2+^. In this pH range, the carboxyl groups in PAAS were more protonated, thus having a lower complexation capacity for metal ions [40]. At pH > 5, Cu^2+^ and Ni^2+^ in the form of Cu(OH)^+^, Cu₂(OH)_2_^2+^, Cu(OH)_2_, Cu_3_(OH)₄^2+^, Ni(OH)^+^, Ni(OH)₂, Ni(OH)_3_^−^, etc., were found [21,29]. At the same time, PAAS is less protonated and can form complexes with most metal ions in the hydroxide form in the solution [41].

#### 3.5.2. Morphological Analysis of Tartaric Acid and Sodium Citrate

**Figure 9:**
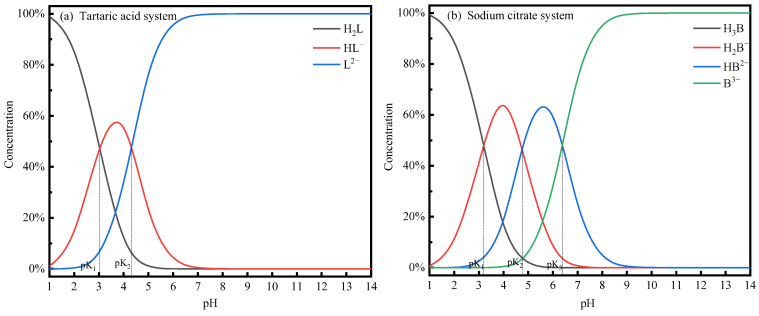
Distribution of components of tartaric acid and sodium citrate solutions.

Under different pH conditions, tartaric acid and sodium citrate are hydrolyzed to different degrees, resulting in different ligands. Figure 9 shows the distribution of solution components of tartaric acid and sodium citrate at different pH values.

From Figure 9a, it can be seen that in the pH range of 1–3, tartaric acid has dissociated a part of HL^−^. This part may be able to complex with metal ions [42] to a certain extent. In this pH range, PAAS is still highly protonated and poorly complexes with metal ions [43]. At this time, part of the Cu^2+^ and Ni^2+^ pass through the membrane pore via HL^−^, while another part directly passes through the membrane pore. The retention rate of PAAS for Cu^2+^ and Ni^2+^ is 0. In the pH range of 3–5, in the tartaric acid system, H_2_L, HL^−^, and L^2−^ exist simultaneously. The degree of protonation of PAAS is reduced. HL^−^, L^2−^, and PAAS can, to a certain extent, be with Cu^2+^ and Ni^2+^. There may be competition for the complexation of Cu^2+^ and Ni^2+^ complexed to different ligands, resulting in the phenomenon that the difference in the retention rate of PAAS for Cu^2+^ and Ni^2+^ increases. In the pH > 5 range, most of the tartaric acid system is L^2−^ and at the same time, the degree of protonation continues to decrease, and the ability to complex with metal ions continues to increase. At this time, most of the Cu^2+^ and Ni^2+^ complexed with PAAS are retained by the ultrafiltration membrane, and the difference in the retention rate of PAAS for Cu^2+^ and Ni^2+^ becomes 0%.

As can be seen from Figure 9b, in the pH range of 1–3, sodium citrate has hydrolyzed a part of H_2_B^−^. H_2_B^−^ may be able to complex with metal ions to a certain extent. In this pH range, PAAS is still highly protonated and has poor complexation with metal ions. At this time, a part of the Cu^2+^ and Ni^2+^ were complexed by H_2_B^−^ through the membrane pore, and another part of the Cu^2+^ and Ni^2+^ passed directly through the membrane pore. As a result, the retention rate of PAAS on Cu^2+^ and Ni^2+^ was 0. In the pH range of 3–5, H_3_B, H_2_B^−^, HB^2−^, and B^3−^ co-exist in the sodium citrate system [44]. The degree of protonation of PAAS is reduced, and H_2_B^−^, HB^2−^, B^3−^, and PAAS all have poor complexation with metal ions. B^3−^ and PAAS can complex with Cu^2+^ and Ni^2+^ to a certain extent. There may be competition for the complexation of Cu^2+^ and Ni^2+^ with different ligands, resulting in the phenomenon of the increasing difference in the PAAS retention rate on Cu^2+^ and Ni^2+^. In the pH > 5 range, most of the sodium citrate system is HB^2−^ and B^3−^. Meanwhile, the degree of PAAS protonation continues to decrease, and its complexation ability with metal ions continues to increase. At this time, most of the Cu^2+^ is retained by PAAS complexation, while Ni^2+^ is retained by HB^2−^ and B^3−^ and passes through the ultrafiltration membrane. This results in the phenomenon of the increasing difference in the PAAS retention rate on Cu^2+^ and Ni^2+^.

At pH = 5, the hydrolysis of sodium citrate generated fewer types of ligands that could be complexed with metal ions. Also, for the same amount of sodium citrate, there were more carboxyl groups compared to tartaric acid. Consequently, adding a lower concentration of sodium citrate was sufficient to improve the separation of metal ions. This is in line with the results shown in Figure 4 and Figure 5.

### 3.6. Infrared Spectral Analysis

**Figure 10:**
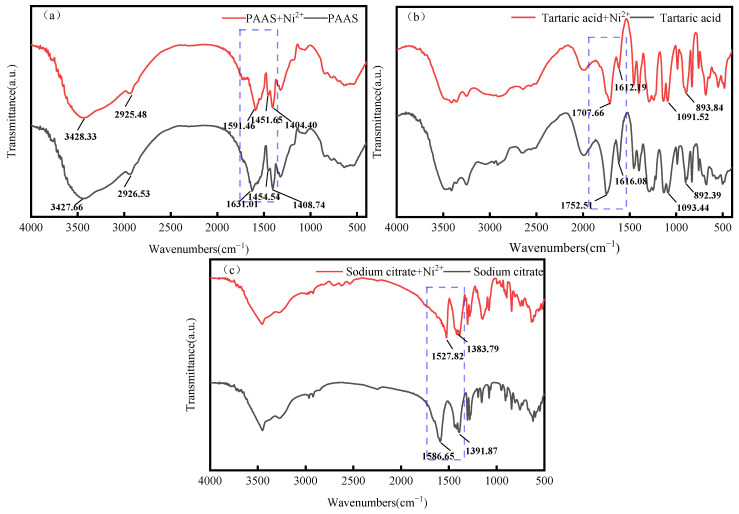
FTIR spectra of samples: (**a**) PAAS and PAAS+Ni^2+^; (**b**) tartaric acid and tartaric acid+Ni^2+^; and (**c**) sodium citrate and sodium citrate+Ni^2+^.

The FTIR spectra of PAAS are presented in Figure 10. It shows an O-H telescopic vibration absorption peak at 3427.66 cm^−1^. Additionally, the symmetric stretching vibration absorption peak of -CH_2_- is at 2926.53 cm^−1^, and the asymmetric and symmetric stretching vibration peaks of C=O in -COO- are near 1631.01 cm^−1^ and 1454.54 cm^−1^, respectively. These are the characteristic peaks of PAAS [45]. As can be observed in Figure 10a, 1631.01 cm^−1^ and 1454.54 cm^−1^ are, respectively, the characteristic peaks of PAAS. After the addition of Ni^2+^, the carboxyl (C=O) stretching vibration peak of PAAS at 1631.01 cm^−1^ shifted to 1591.46 cm^−1^, with a shift of 39.55 cm^−1^. This indicates that PAAS interacts with Ni^2+^ through chemical bonding.

#### 3.6.1. Analysis of Complexation Behavior of Ni^2+^ in Systems of Tartaric Acid and Sodium Citrate

The FTIR spectra of tartaric acid revealed that the peaks at 1752.51 cm^−1^, 1616.08 cm^−1^, 1093.44 cm^−1^, and 892.39 cm^−1^ were absorption peaks resulting from the carboxyl group (C=O) and (O-H) vibrations on the carboxyl group, thereby indicating the existence of the carboxyl group in tartaric acid. As can be observed from Figure 10b, the peaks near 1752.51 cm^−1^ and 1616.08 cm^−1^ are characteristic peaks of tartaric acid [46]. After the addition of NiSO_4_, the peak of tartaric acid at 1752.51 cm^−1^ shifted to 1707.66 cm^−1^, with a shift of 48.55 cm^−1^. This phenomenon indicates that tartaric acid interacts with Ni^2+^ through chemical bonding. As the characteristic peaks of tartaric acid shifted more after its interaction with NiSO_4_, it can be hypothesized that the complex formed by tartaric acid and Ni^2+^ is more stable than the one formed by PAAS and Ni^2+^. Therefore, it can be hypothesized that the complexes formed by tartaric acid with Ni^2+^ are more stable than those formed by PAAS with Ni^2+^. Consequently, when tartaric acid is added to the system where PAAS is in complex with Ni^2+^, the system is more inclined to generate a complex of tartaric acid with Ni^2+^. From Figure 10c, 1586.65 cm^−1^ and 1391.87 cm^−1^ are, respectively, the characteristic peaks of sodium citrate [46]. After the addition of NiSO_4_, the peak at 1586.65 cm^−1^ of sodium citrate shifted to 1527.82 cm^−1^ by 58.83 cm^−1^, indicating that sodium citrate interacts with Ni^2+^ through chemical bonding. Since the characteristic peaks of calcium citrate shifted more after interacting with NiSO_4_, it is assumed that the complex formed by sodium citrate and Ni^2+^ is more stabilized than that formed by PAAS and Ni^2+^. Therefore, when sodium citrate is added to the system of PAAS and Ni^2+^, the system tends to produce complexes of sodium citrate and Ni^2+^.

#### 3.6.2. Analysis of Complexation Behavior of Cu^2+^ in Tartaric Acid and Sodium Citrate Systems

**Figure 11:**
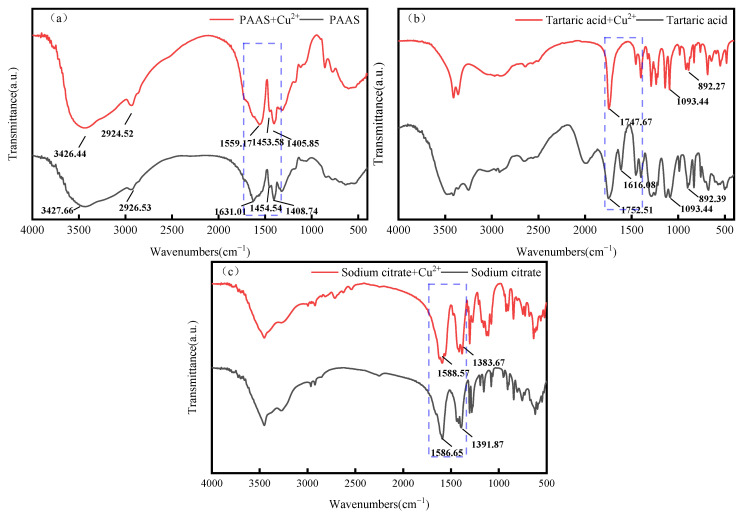
FTIR spectra of samples: (**a**) PAAS and PAAS+Cu^2+^; (**b**) tartaric acid and tartaric acid+Cu^2+^; and (**c**) sodium citrate and sodium citrate+Cu^2+^.

As can be seen from Figure 11a, upon the addition of Cu^2+^, the carboxyl (C=O) stretching vibration peak of PAAS at 1631.01 cm^−1^ shifts to 1559.17 cm^−1^, with a shift of 71.84 cm^−1^. This is similar to the results of Huo Junda’s study [47]. This suggests that PAAS interacts with Cu^2+^ using chemical bonding.

As can be seen in Figure 11b, upon the addition of Cu^2+^, the carboxyl group (C=O) stretching vibration peak of tartaric acid at 1752.51 cm^−1^ shifted to 1747.67 cm^−1^, with a shift of 4.84 cm^−1^. This indicates that tartaric acid interacts with Cu^2+^ through chemical bonding. Since the characteristic peak of PAAS shifted more after interacting with Cu^2+^, it was hypothesized that the interaction of PAAS with Cu^2+^ is more stable compared to that formed by tartaric acid and Cu^2+^. Therefore, the addition of tartaric acid to the system where PAAS is complexed with Cu^2+^ tends to generate the complex of PAAS with Cu^2+^ in the system. As depicted in Figure 11c, upon the addition of Cu^2+^, the carboxyl (C=O) stretching vibration peak of sodium citrate at 1586.65 cm^−1^ shifts to 1588.57 cm^−1^, with a shift of 1.92 cm^−1^. This indicates that sodium citrate interacts with Cu^2+^ through chemical bonding. Moreover, as the characteristic peak of PAAS undergoes a more significant shift after interacting with Cu^2+^, it suggests a preference within the system for the generation of complexes of PAAS with Cu^2+^.

### 3.7. XPS Analysis

**Figure 12:**
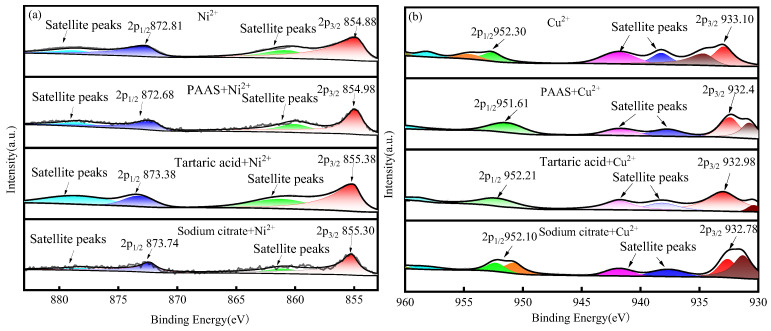
Fitted peaks of fine spectra for metal ions: (**a**) Ni2p fine spectrum; (**b**) Cu2p fine spectrum.

Figure 12 presents the peaks of the metal ions in each system, along with the fine spectra and binding energies of Ni2p and Cu2p.

The Ni2p1/2 and Ni2p3/2 of Ni^2+^ in NiSO_4_ are located at 872.81 eV and 854.88 eV, respectively, which are consistent with the literature reports [48]. After adding PAAS, the Ni2p characteristic peak of Ni^2+^ shifts due to the complexation of Ni^2+^ by PAAS. Similarly, after adding tartaric acid and sodium citrate, the Ni2p characteristic peak shifts because of the complexation of Ni^2+^ by them. The shift in the Ni2p characteristic peak after PAAS complexation with Ni^2+^ is approximately 0.1 eV, which is much smaller than that after the complexation of tartaric acid and Ni^2+^. After the addition of tartaric acid and sodium citrate, the Ni2p characteristic peak of Ni^2+^ shifts due to complexation. Meanwhile, the Ni2p characteristic peak of PAAS complexed with Ni^2+^ shifts by about 0.1 eV, significantly smaller than the Ni2p characteristic peak of tartaric acid complexed with Ni^2+^ (0.5 eV) and the Ni2p characteristic peak of sodium citrate complexed with Ni^2+^ (0.42 eV). This indicates that the stability of the Ni^2+^ action of tartaric acid and sodium citrate is greater than that of the Ni2p characteristic peak of the PAAS complexed with Ni^2+^. Therefore, in the Ni^2+^ system, XPS can show that the addition of tartaric acid to the complexation system of PAAS with Ni^2+^ tends to generate more stable sodium citrate–Ni and tartaric acid–Ni complexes. This can be mutually verified with the results of Figure 4 and Figure 6, where the addition of a certain concentration of tartaric acid/sodium citrate resulted in Ni^2+^ being complexed by tartaric acid/sodium citrate through the membrane pores, and the retention rate of Ni^2+^ by PAAS decreased.

The Cu2p1/2 and CU2p3/2 of Cu^2+^ in CuSO_4_ were, respectively, located at 952.30 eV and 933.01 eV, which is basically in line with the literature reports [49]. After the addition of PAAS, the Cu2p peak of Cu^2+^ shifted due to the complexation of PAAS with Cu^2+^. Similarly, after the addition of tartaric acid and sodium citrate, the Cu2p peak of Cu^2+^ also shifted because of the complexation by these substances. The displacement of the Cu2p peak of the PAAS complexed with Cu^2+^ is approximately 0.7 eV, which is significantly larger than that of the Cu2p peak of the tartaric acid complexed with Cu^2+^ (0.12 eV) and the Cu2p peak of the sodium citrate complexed with Cu^2+^ (0.32 eV). This implies that the stability of the Cu^2+^ effect of tartaric acid and sodium citrate is lower than that of the Cu^2+^ effect of PAAS. Therefore, in the Cu^2+^ system, XPS can show that the addition of tartaric acid and sodium citrate to the complexation system of PAAS with Cu^2+^ tends to generate more stable PAAS-Cu complexes. This can be mutually verified by the results in Figure 4 and Figure 6. After adding a certain concentration of tartaric acid/sodium citrate, Cu^2+^ was retained above the membrane through PAAS complexation, and the change in the PAAS- retained Cu^2+^ was small.

## 4. Conclusions

This study focuses on the effect of tartaric acid and sodium citrate on the separation and recovery of Cu^2+^ and Ni^2+^ by PAAS, as well as the characterization of complexes formed by tartaric acid, sodium citrate, and PAAS with metal ions.

Under the condition of no additions of tartaric acid and sodium citrate, the difference in the retention rate of Cu^2+^ and Ni^2+^ by PAAS can only reach 45.36% under the optimum separation condition. However, after adding a certain concentration of tartaric acid and sodium citrate, the retention rate difference in PAAS for Cu^2+^ and Ni^2+^ can be expanded to 80.36% and 58.84%, respectively, under the optimal separation conditions, significantly improving the separation effect of Cu^2+^ and Ni^2+^.

The graphs of the components of the solutions of Cu^2+^, Ni^2+^, tartaric acid, and sodium citrate indicate that at pH = 5, compared with tartaric acid, sodium citrate can hydrolyze more types of ligands, and there is a competitive complexation of PAAS and tartaric acid/sodium citrate with Cu^2+^ and Ni^2+^ in the system. Infrared and XPS plot results show that PAAS, tartaric acid/sodium citrate, Cu^2+^, and Ni^2+^ have different complexation abilities, which changes the complexation objects of Cu^2+^ and Ni^2+^, thus improving the separation effect of Cu^2+^ and Ni^2+^ and being more helpful for the subsequent recovery of Cu^2+^ and Ni^2+^.

As far as cost is concerned, the actual plating wastewater has a complex composition and may contain small amounts of sodium citrate and tartaric acid, which are widely available and inexpensive. Therefore, subsequent use costs can be saved. Besides improving the recovery efficiency of metal ions in electroplating wastewater, in terms of operational difficulty, only adding tartaric acid/sodium citrate and adjusting the pH are required, and the subsequent discharge will not have a significant impact on the environment. Therefore, this work has the potential to be applied to the treatment of plating wastewater containing low concentrations of Cu^2+^ and Ni^2+^. Compared with other technologies (e.g., chemical precipitation, adsorption), this technology is less expensive and easier to operate.

## Figures and Tables

**Table 1:** List of ultrafiltration membrane models.

Membrane Type	Material	MWCO/kDa	Membrane Area/m^2^
Tubular membrane	PES	20~50	0.2
Flat membrane	PES	20~50	0.01

**Table 2:** List of reagents used in the test.

Chemicals	Purity	Manufacturer	Use
H_2_SO_4_	>95%	Kemiou	Adjustment of pH
NaOH	100%	Kemiou	Adjustment of pH
NiSO_4_∙6H_2_O	>98.5%	Damao	Simulate wastewater
CuSO_4_	>99%	Damao	Simulate wastewater
Tartaric acid	>98%	Zhiyuan	Complexing agent
Sodium citrate	>98%	Guang fu Fine Chemical	Complexing agent
HNO_3_	>95%	SINOPHARM	Concentration analysis
Cu^2+^ standard solution	1000 μg/mL	GBTC	Concentration analysis
Ni^2+^ standard solution	1000 μg/mL	GBTC	Concentration analysis

## Data Availability

The original contributions presented in the study are included in the article and Supplementary Materials; further inquiries can be directed to the corresponding author.

## Supplementary Materials

The following supporting information can be downloaded at: https://www.mdpi.com/article/10.3390/membranes14110240/s1, Figure S1: Diagram of the pretreatment test setup.; Figure S2: PAAS, tartaric acid, sodium citrate Tyndall effect.; Figure S3: Change in membrane flux.
