# Supplementary material for: Separation of Copper and Nickel Metal Ions from Electroplating Wastewater by Ultrafiltration with Tartaric Acid and Sodium Citrate Reinforced Sodium Polyacrylate Complexation
Source: Membranes (Basel). 2024 Nov 14;14(11):240. doi: 10.3390/membranes14110240 (PMC11596544; doi:10.3390/membranes14110240)
Supplement: Supplementary file 1 [file membranes-14-00240-s001.zip › membranes-3281604-supplementary.pdf]

Supplementary materials

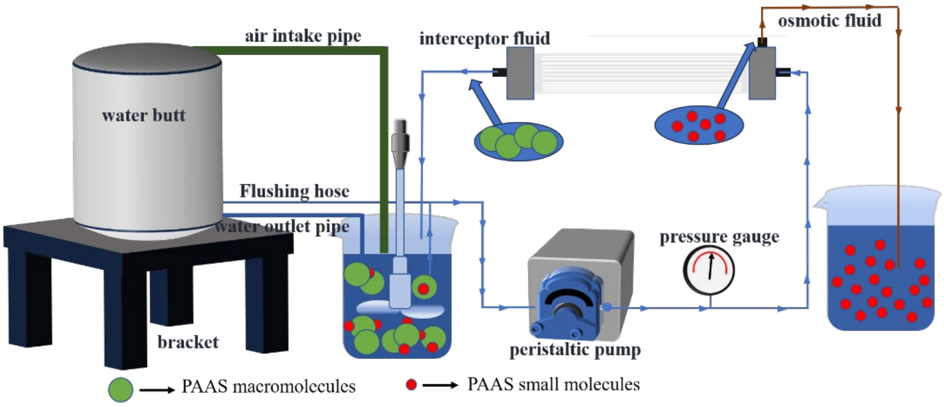

Figure S1. Diagram of the pretreatment test setup.

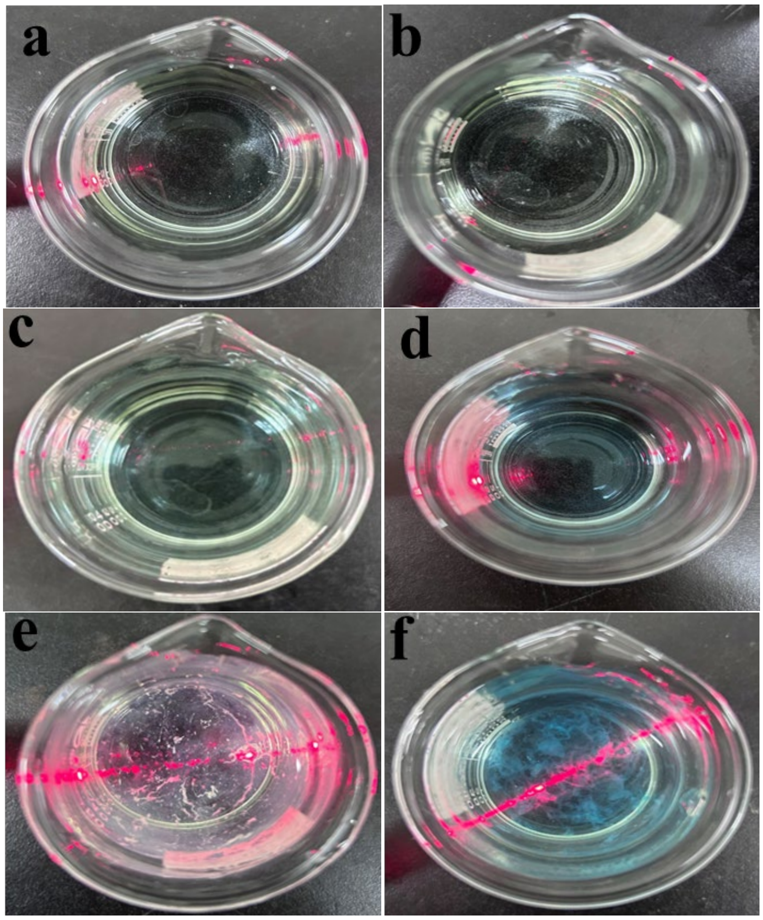

Figure S2. PAAS, tartaric acid, sodium citrate Tyndall effect.

(a) tartaric acid+Ni^{2+}, (b) tartaric acid+Cu^{2+},

(c) sodium citrate +Ni^{2+}, (d) sodium citrate +Cu^{2+},

(e) PAAS+Ni^{2+}, (f) PAAS+Cu^{2+}.

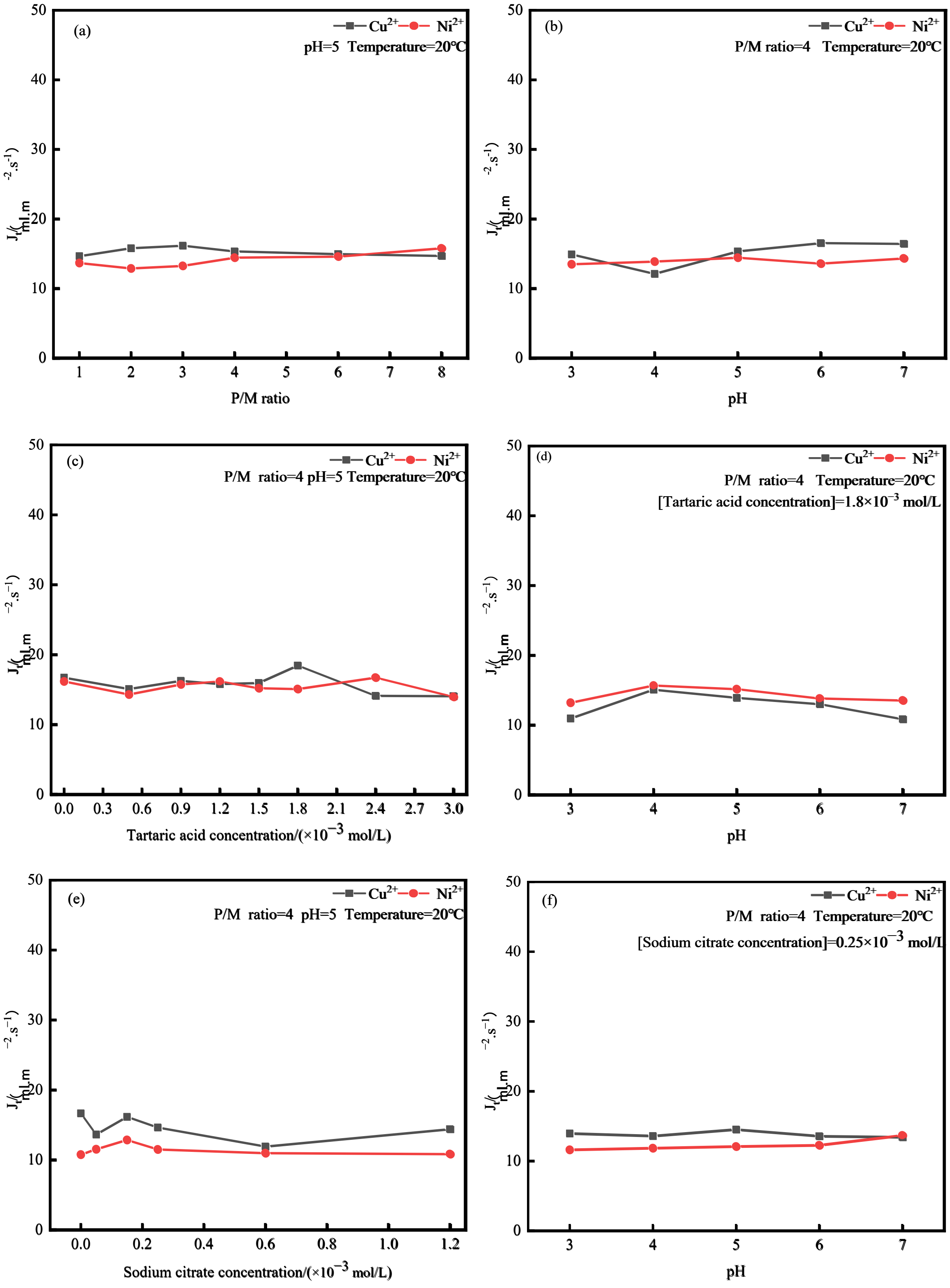

Figure S3. Change in membrane flux.

(a) Effect of pH on the membrane flux.

(b) Effect of P/M ratio on the membrane flux.

(c) Effect of tartaric acid concentration on the membrane flux.

(d) Effect of pH on the membrane flux after the addition of tartaric acid.

(e) Effect of sodium citrate concentration on membrane flux.

(f) Effect of pH on the membrane flux after the addition of sodium citrate.
